# Whole-Genome Approach to Assessing Human Cytomegalovirus Dynamics in Transplant Patients Undergoing Antiviral Therapy

**DOI:** 10.3389/fcimb.2020.00267

**Published:** 2020-06-15

**Authors:** Nicolás M. Suárez, Emily Blyth, Kathy Li, Tina Ganzenmueller, Salvatore Camiolo, Selmir Avdic, Barbara Withers, Silvia Linnenweber-Held, Wilfried Gwinner, Akshay Dhingra, Albert Heim, Thomas F. Schulz, Rory Gunson, David Gottlieb, Barry Slobedman, Andrew J. Davison

**Affiliations:** ^1^MRC-University of Glasgow Centre for Virus Research, Glasgow, United Kingdom; ^2^Westmead Institute for Medical Research, Sydney, NSW, Australia; ^3^Faculty of Medicine and Health, The University of Sydney, Sydney, NSW, Australia; ^4^Blood and Bone Marrow Transplant Unit, Westmead Hospital, Sydney, NSW, Australia; ^5^Sydney Cellular Therapies Laboratory, Westmead Hospital, Sydney, NSW, Australia; ^6^Institute for Medical Virology, University Hospital Tübingen, Tübingen, Germany; ^7^Institute of Virology, Hannover Medical School, Hanover, Germany; ^8^Department of Nephrology, Hannover Medical School, Hanover, Germany; ^9^Public Health Agency of Lower Saxony, Hanover, Germany; ^10^German Center for Infection Research, Hanover, Germany; ^11^West of Scotland Specialist Virology Centre, Glasgow Royal Infirmary, Glasgow, United Kingdom; ^12^Discipline of Infectious Diseases and Immunology, Faculty of Medicine and Health, Charles Perkins Centre, The University of Sydney, Sydney, NSW, Australia

**Keywords:** human cytomegalovirus, transplantation, genome sequence, target enrichment, multiple-strain infection, antiviral therapy, resistance mutation

## Abstract

Human cytomegalovirus (HCMV) is the most frequent cause of opportunistic viral infection following transplantation. Viral factors of potential clinical importance include the selection of mutants resistant to antiviral drugs and the occurrence of infections involving multiple HCMV strains. These factors are typically addressed by analyzing relevant HCMV genes by PCR and Sanger sequencing, which involves independent assays of limited sensitivity. To assess the dynamics of viral populations with high sensitivity, we applied high-throughput sequencing coupled with HCMV-adapted target enrichment to samples collected longitudinally from 11 transplant recipients (solid organ, *n* = 9, and allogeneic hematopoietic stem cell, *n* = 2). Only the latter presented multiple-strain infections. Four cases presented resistance mutations (*n* = 6), two (A594V and L595S) at high (100%) and four (V715M, V781I, A809V, and T838A) at low (<25%) frequency. One allogeneic hematopoietic stem cell transplant recipient presented up to four resistance mutations, each at low frequency. The use of high-throughput sequencing to monitor mutations and strain composition in people at risk of HCMV disease is of potential value in helping clinicians implement the most appropriate therapy.

## Introduction

Despite continuous advances in diagnostics and therapy, human cytomegalovirus (HCMV) remains the most frequent opportunistic viral infection following transplantation, contributing significantly to patient morbidity, and mortality (Boeckh and Ljungman, 2009). The risk of developing HCMV disease after transplantation depends on a number of host factors, including the HCMV serological status of the donor and the recipient, the type of transplant and the level of immunosuppression. In addition, viral factors are associated with poorer outcomes, including the selection of mutants resistant to antiviral drugs and the occurrence of infections involving multiple HCMV strains (Wu et al., 2010; Lisboa et al., 2012).

Viral factors are usually addressed by analyzing individual HCMV genes by polymerase chain reaction (PCR) and Sanger sequencing. Resistance mutations can be screened in the genes responsible for antiviral drug activity (typically UL54 encoding the viral DNA polymerase, and UL97 encoding a phosphotransferase), whereas multiple-strain infections may be detected by genotyping hypervariable genes (commonly UL73 encoding glycoprotein N [gN], UL74 [gO], and UL55 [gB]). This strategy involves an independent assay for each gene analyzed, and its sensitivity is generally limited to the detection of subpopulations exceeding 20% of the total viral population (Schuurman et al., 1999; Sahoo et al., 2013).

Monitoring the complexity of viral populations and the dynamics of resistance mutations is critical to understanding the evolutionary mechanisms operating on the virus during antiviral selection. This is also clinically relevant because the presence of cells infected with multiple strains provides an opportunity for the virus to recombine (Rasmussen et al., 2003), which might lead to an increase of viral strain pathogenicity. Moreover, the presence of drug-resistant populations at low frequencies (<5%) during the early stages of antiviral treatment may signal eventual treatment failure when these populations come to predominate at later stages (Chou et al., 2014; Houldcroft et al., 2016). Indeed, continuation with the antiviral drug to which resistance has developed can lead to the development of additional resistance mutations (Lurain and Chou, 2010). In contrast, therapy modification can accelerate viral clearance (Castagnola et al., 2004). Thus, rapid, comprehensive and sensitive molecular diagnostic approaches would be beneficial for assisting clinicians in improving the management of HCMV infections.

The application of commercial target enrichment methods coupled with the use of high-throughput sequencing (HTS) has dramatically surpassed the sensitivity of PCR methods for characterizing HCMV in clinical material (Hage et al., 2017; Cudini et al., 2019; Suárez et al., 2019a,b). This approach has the great advantage of allowing resistant populations and strain numbers to be assessed across the whole viral genome in a single assay. Studies employing analysis of individual HCMV genes by PCR and HTS to detect antiviral resistance mutations in HCMV infections have been reported (Sahoo et al., 2013; Chou et al., 2014; Guermouche et al., 2020), but none has applied a whole-genome approach. Moreover, sophisticated bioinformatic tools have recently been developed to facilitate accurate interrogation of HTS data for the presence of multiple HCMV strains (Suárez et al., 2019a,b). Here, we used HTS and HCMV-adapted bioinformatics resources to assess the dynamics of HCMV viral populations in 11 transplant recipients undergoing antiviral therapy.

## Materials and Methods

### Sample Collection

**Figure 1:**
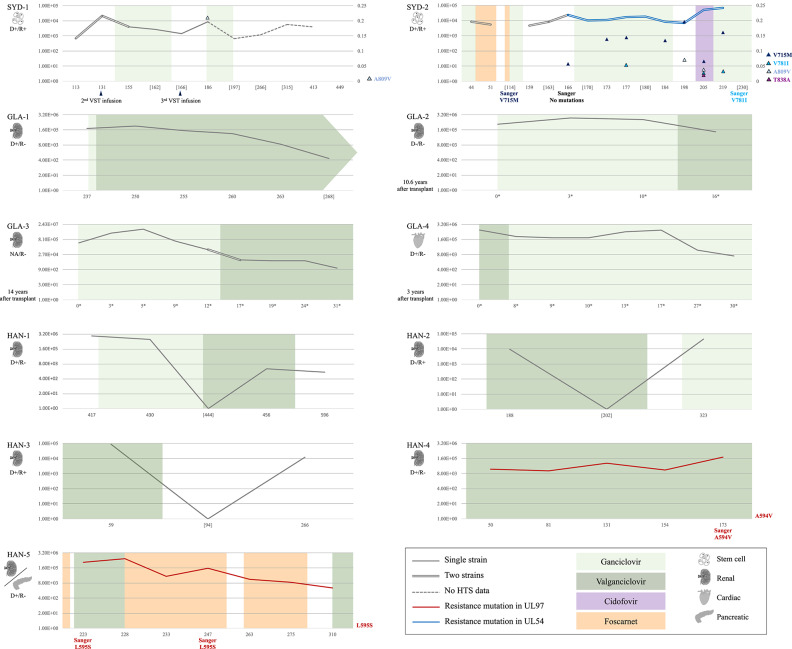
Information on the cohort (*n* = 11): clinical data, level of viremia, and presence of multiple-strain infections and resistance mutations. Data on type of transplant and HCMV serological status (positive, +; and negative, –) of the donor (D) and recipient (R) is given on the left side of each panel (NA, not available). Viral loads (traces) are expressed in log_10_ [IU/ml] (left Y-axis). Dashed traces indicate lack of HTS data for the corresponding time points. Chronological data (X-axis) are represented in days after transplant, or in days after reactivation (*) if the time between transplant and sample collection was >3 years (patients GLA-2, GLA-3, and GLA-4). Sample collection points lacking corresponding HTS data are shown in square brackets. Sanger sequencing results and application of virus-specific T cell (VST) infusions are indicated at the corresponding time points under the X-axis. Application and duration of antiviral therapy are represented by colored blocks (light green, ganciclovir; dark green, valganciclovir; purple, cidofovir; and orange, foscarnet). Single-strain and multiple-strain infections are represented by single and double traces, respectively. Red traces indicate the presence of fixed resistance mutations (frequency 100%; A594V in patient HAN-4 and L595S in patient HAN-5) in the UL97 gene, and a blue trace indicates the presence of resistance mutations in the UL54 gene (patient SYD-2). In patients SYD-1 and SYD-2, the right Y-axis shows the frequency of low-level resistance mutations (frequency >2%), which are represented by colored triangles embedded in the panels. The resistance mutations detected in each of these patients are indicated on the right side of the panels.

Plasma (*n* = 42) and whole blood (*n* = 20) samples from confirmed HCMV-infected transplant recipients (nine solid organ transplants and two allogeneic hematopoietic stem cell transplants) were collected longitudinally during episodes of viremia. They were collected at times following transplantation or after HCMV reactivation ranging from 16 to 336 days, and had viral loads ranging from 6.94 × 10^2^ to 8.13 × 10^6^ HCMV international units per ml (IU/ml) of plasma or whole blood. All transplant patients underwent antiviral therapy with valganciclovir, ganciclovir, foscarnet or cidofovir alone or in combination with T cell therapy (Table 1 and Figure 1). Samples were collected with the approval of the University of Sydney Human Research Ethics Committee (reference 2014/440), the National Health Service research Scotland Greater Glasgow and Clyde Biorepository (reference 405) and the Institutional review boards of Hannover Medical School (reference 2527-2014).

**Table 1:** Clinical information on transplant patients.

**Patient ID**	**Type of transplant**	**Specimen**	**HCMV D/R serostatus**	**Time span from first to last sample (days)**	**Number of sequential samples**	**Antiviral regime**
SYD-1	Allogeneic hematopoietic stem cell	Plasma	D+/R+	336	6	GCV, HCMV-T
SYD-2	Allogeneic hematopoietic stem cell	Plasma	D+/R+	175	10	FSC, GCV, CDV
GLA-1	Renal	Plasma	D+/R–	31	5	GCV, vGCV
GLA-2	Renal	Plasma	D–/R–	16	4	GCV, vGCV
GLA-3	Renal	Plasma	NA/R–	31	9	GCV, vGCV
GLA-4	Cardiac	Plasma	D+/R–	30	8	GCV, vGCV
HAN-1	Renal	Blood	D+/R–	179	4	GCV, vGCV
HAN-2	Renal	Blood	D–/R+	135	2	GCV, vGCV
HAN-3	Renal	Blood	D+/R+	207	2	vGCV
HAN-4	Renal	Blood	D+/R–	123	5	vGCV
HAN-5	Renal/Pancreatic	Blood	D+/R–	87	7	FSC, vGCV

*D, donor; R, recipient; HCMV-T, HCMV-specific T cells; CDV, cidofovir; GCV, ganciclovir; vGCV, valganciclovir; FSC, foscarnet; NA, data not available*.

### DNA Extraction, Genomic Library Preparation, and DNA Sequencing

Total DNA was extracted from 200 μl of clinical material using the QIAamp MinElute virus spin kit for plasma samples and the QIAamp DNA blood mini kit for whole blood samples (QIAGEN, Crawley, UK). An aliquot of 50 μl of extracted DNA was sheared acoustically using an LE220 sonicator (Covaris, Woburn, MA, USA), aiming to achieve an average fragment size of 500 bp. The fragmented DNA was prepared for sequencing using a library preparation kit (KAPA Biosystems, London, UK) and following the SureSelect^XT^ version 1.7 target enrichment system (Agilent Technologies, Santa Clara, CA, USA) as described previously (Hage et al., 2017; Suárez et al., 2019b). Libraries were indexed using ultrapure (TruGrade) oligonucleotides (Integrated DNA Technologies, Leuven, Belgium), and loaded onto a NextSeq (Illumina, San Diego, CA, USA) DNA sequencer to generate 2 × 150 bp paired-ended reads.

### Genome Assembly and Data Analysis

Sequencing data quality was enhanced by removing low-quality regions from the reads using Trim Galore (http://www.bioinformatics.babraham.ac.uk/projects/trim_galore/) (length = 21, quality = 10, and stringency = 3). The remaining paired-ended reads were screened for the presence of multiple HCMV strains using a method based on detecting signatures unique to the individual genotypes of hypervariable genes (Suárez et al., 2019b). Briefly, multiple strains were assigned if at least two genotypic signatures were found for at least two of the 12 hypervariable genes tested, and if these represented ≥2% of the total number of reads having any genotypic signature of these genes. In the datasets reflecting the presence of a single strain (i.e., presenting single genotypic signatures in at least ten hypervariable genes), the paired-ended reads were assembled *de novo* using SPAdes 3.5.0 (Bankevich et al., 2012) after removing reads mapping to the Genome Reference Consortium Human Reference 38 sequence (http://genome.ucsc.edu/) using Bowtie2 v.2.2.6 (Langmead and Salzberg, 2012). The contigs were then assembled into genomes using Scaffold_builder (Silva et al., 2013) and the publicly available sequence of HCMV reference strain Merlin (GenBank accession no. AY446894.2). Subsequently, the reads were mapped to the respective assembled genome using Bowtie2, and the alignments were inspected visually using Tablet (Milne et al., 2013). Drug resistance mutations within the antiviral target genes (UL54 and UL97) were screened at two frequency levels: (1) at high frequency by interrogating the UL54 and UL97 gene consensus sequences of the assembled genomes using the online resource Mutation Resistance Analyzer (Chevillotte et al., 2010), which employs HCMV strain TB40/E as reference, and (2) at low frequency by interrogating high-quality datasets (criteria are defined in Results) mapped to corresponding assemblies using LoFreq (Wilm et al., 2012). The latter analysis was conducted using a conservative multi-step filtering approach aimed at minimizing the detection of false positive variants. Briefly, read quality was enhanced using Trim Galore with default parameters, trimmed reads were deduplicated (i.e., duplicated reads were removed) using FastUniq (Xu et al., 2012), and read quality was further improved using PRINSEQ (Schmieder and Edwards, 2011) (minimum quality mean = 25, trimming quality left = 30, trimming quality window = 5, trimming quality step = 1, minimum length = 80, and trimming 3' Ns = 20). Imperfect poly-G tracts located at the 3' ends of reads were trimmed when a segment of 40 nucleotides contained >30 G residues. Filtered sequencing reads were mapped to their respective assemblies using Bowtie2 in end-to-end alignment mode. Reads were further deduplicated on the basis of alignment coordinates using Picard (http://broadinstitute.github.io/picard/). Variants with a quality score of ≥30 were called from the deduplicated alignments.

### Data Deposition

Datasets were purged of human reads and deposited in the European Nucleotide Archive (ENA; project no. PRJEB36759), and the annotated consensus genome sequences were deposited in GenBank (accession nos. MT044476-MT044485).

## Results

### Quality Assessment of Sequencing Libraries and Enumeration of Strains

A total of 62 sequencing libraries from 11 transplant patients (2–10 samples per patient) were generated, with input HCMV values ranging from 39 to 1.6 × 10^6^ IU per library. Sequencing yielded 7.6 × 10^6^ to 24.5 × 10^6^ trimmed reads per sample, with 0–87% mapping to the HCMV reference strain Merlin genome (Table S1, rows 14 and 15). When reads were mapped to the strain Merlin genome, the average sequencing depth ranged from 5 to 12,016 reads/nucleotide (nt), covering between 35 and 100% of the Merlin reference genome (Table S1, rows 16 and 17).

**Figure 2:**
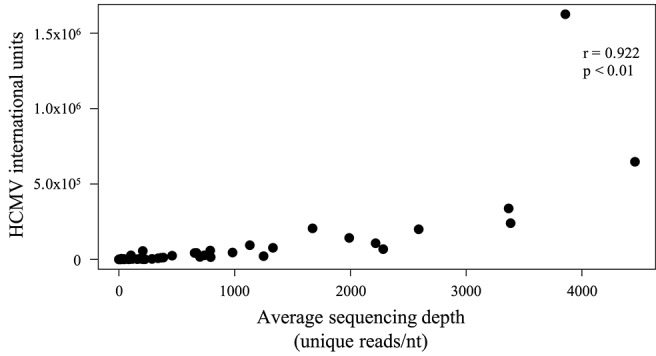
Scatter plot showing the effect of the number of genome copies used to make a sequencing library on the diversity of the sequencing data obtained. The number of genome copies (IU) is represented on the Y-axis, and the average sequencing depth (reads/nt) of unique HCMV reads is represented on the X-axis. Unique reads were identified as described in Methods. A significant (*p* < 0.01) positive correlation (Spearman) was noted.

To examine library fragment diversity, the average sequencing depth of reads mapped to the strain Merlin genome was calculated after removing duplicates. The average sequencing depth of unique reads ranged from 0.001 (low diversity) to 4,458 (high diversity) reads/nt (Table S1, row 18), and generally reflected the number of HCMV genome copies in the input material (Figure 2).

Using the previously published method for distinguishing viral strains by monitoring the genotypes of hypervariable genes (Suárez et al., 2019b), multiple HCMV strains were detected in 15 datasets derived from three patients (SYD-1, SYD-2, and GLA-3). Multiple strains were detected in 4/6 datasets derived from patient SYD-1, 10/10 datasets derived from patient SYD-2, and 1/9 datasets derived from patient GLA-3 (Table 2).

**Table 2:** Genotyping of 12 hypervariable genes and estimation of strains numbers.

**Dataset ID**	**Genes**												**Strains (no.)**
	**RL5A**	**RL6**	**RL12**	**RL13**	**UL1**	**UL9**	**UL11**	**UL73**	**UL74**	**UL120**	**UL146**	**UL139**	
SYD-1_T01	1	**1,2**	1A	1**,8**	**1,8**	**1,9**	**1,3**	2	**2B,5**	**1B,2A**	12	3	**2**
SYD-1_T02	1	1	1A	1	1	1	3	**2,4D**	2B	**1B,2A**	12	3	**2**
SYD-1_T03	1	1	1A	1	1	1	3	2	2B	1B	12	3	1
SYD-1_T04	1	1	1A	1	1	1	3	2	2B	1B	12	3	1
SYD-1_T05	1	**1,2**	**1A,8**	1	**1,8**	1	3	**2,4D**	5	**1B,2A**	12	4	**2**
SYD-1_T06	1	**1,2**	**1A,8**	1**,8**	**1,8**	**1,9**	**1,3**	**2,4D**	**2B,5**	**1B,2A**	12	3**,4**	**2**
SYD-2_T01	1	3	4B	4B	4	**4,9**	**1,5**	4B	4	**1B,2A**	7	4	**2**
SYD-2_T02	1	3	4B	4B	4	**4,9**	5	4B	4	**1B,2A**	———	**2,4**	**2**
SYD-2_T03	1	**3,4**	4B	**1,4B**	4	9	5	4B	4	**1B,2A**	———	4	**2**
SYD-2_T04	**1,2**	**3,4**	**1A,4B**	**1,4B**	**1,4**	**4,9**	**1,5**	4B	4	**1B,2A**	**1,7**	**2,4**	**2**
SYD-2_T05	1	**3,4**	**1A,4B**	4B	**1,4**	9	5	4B	4	1B	7	**2,4**	**2**
SYD-2_T06	1	**3,4**	4B	4B	**1,4**	**4,9**	5	4B	4	1B	7	4	**2**
SYD-2_T07	**1,2**	3	4B	4B	4	**4,9**	5	4B	4	1B	7	4	**2**
SYD-2_T08	1	3	4B	**1,4B**	**1,4**	9	**1,5**	**3B,4B**	4	**1B,2A**	**1,7**	4	**2**
SYD-2_T09	1	**3,4**	4B	4B	4	**4,9**	**1,5**	4B	4	**1B,2A**	7	**2,4**	**2**
SYD-2_T10	1	**3,4**	4B	**1,4B**	**1,4**	**4,9**	**1,5**	4B	4	**1B**	**1,7**	**2,4**	**2**
GLA-1_T01	1	3	10	10	10	8	7	3A	1B	3A	1	2	1
GLA-1_T02	1	3	10	10	10	8	7	3A	1B	3A	1	2	1
GLA-1_T03	1	3	10	10	10	8	7	3A	1B	3A	1	2	1
GLA-1_T04	1	3	10	10	10	8	7	3A	1B	3A	1	2	1
GLA-1_T05	1	3	10	10	10	8	7	3A	1B	3A	1	2	1
GLA-2_T01	2	4	2	2	2	1	3	4A	3	2A	14	4	1
GLA-2_T02	2	4	2	2	2	1	3	4A	3	2A	14	4	1
GLA-2_T03	2	4	2	2	2	1	3	4A	3	2A	14	4	1
GLA-2_T04	2	4	2	2	2	1	3	4A	3	2A	14	4	1
GLA-3_T01	2	4	10	10	10	1	4	2	2B	1A	11	2	1
GLA-3_T02	2	4	10	10	10	1	4	2	2B	1A	11	2	1
GLA-3_T03	2	4	10	10	10	1	4	2	2B	1A	11	2	1
GLA-3_T04	2	4	10	10	10	1	4	2	2B	1A	11	2	1
GLA-3_T05	**1,2**	4	**8,10**	10	10	1	**1,4**	2	2B	**1A,4B**	**2,11**	2	**2**
GLA-3_T06	2	**1,4**	10	10	10	1	4	2	2B	1A	11	2	1
GLA-3_T07	2	4	10	10	10	1	4	2	2B	1A	11	2	1
GLA-3_T08	2	**1,4**	10	10	10	1	4	2	2B	1A	11	2	1
GLA-3_T09	2	———	———	———	10	———	**4, 7**	2	2B	———	———	2	1
GLA-4_T01	2	4	6	6	6	7	3	4B	4	3A	1	5	1
GLA-4_T02	2	4	6	6	6	7	3	4B	4	3A	1	5	1
GLA-4_T03	2	4	6	6	6	7	3	4B	4	3A	1	5	1
GLA-4_T04	2	4	6	6	6	7	3	4B	4	3A	1	5	1
GLA-4_T05	2	4	6	6	6	7	3	4B	4	3A	1	5	1
GLA-4_T06	2	4	6	6	6	7	3	4B	4	3A	1	5	1
GLA-4_T07	2	4	6	6	6	7	3	4B	4	3A	1	5	1
GLA-4_T08	2	4	6	6	6	7	3	4B	4	3A	1	5	1
HAN-1_T01	5	1	4A	4A	4	1	1	3B	2A	2A	13	4	1
HAN-1_T02	5	1	4A	4A	4	1	1	3B	2A	2A	13	4	1
HAN-1_T03	———	———	———	———	10	———	———	———	———	———	———	———	1
HAN-1_T04	5	———	———	———	4	1	———	———	———	———	———	———	1
HAN-2_T01	———	2	4A	10	3	9	**———**	———	**———**	**———**	**———**	**———**	1
HAN-2_T02	6	———	4B	———	4	6	1	———	———	———	———	2	1
HAN-3_T01	3	5	3	3	3	2	1	4A	3	**1A,3A**	14	5	1
HAN-3_T02	———	———	3	———	———	———	———	———	———	———	———	———	1
HAN-4_T01	———	1	———	2	2	———	3	**3B,4C**	———	———	———	———	1
HAN-4_T02	———	———	———	———	2	———	———	4C	———	———	———	———	1
HAN-4_T03	3	5	———	2	———	1	3	4C	1C	2A	8	4	1
HAN-4_T04	3	5	———	———	———	1	3	4C	1C	2A	———	4	1
HAN-4_T05	3	5	2	2	2	1	3	4C	1C	2A	8	4	1
HAN-5_T01	1	3	8	8	8	1	1	4D	5	3B	1	3	1
HAN-5_T02	1	3	8	8	8	1	1	4D	5	3B	1	3	1
HAN-5_T03	1	3	8	8	8	1	1	4D	5	3B	1	3	1
HAN-5_T04	1	3	8	8	8	1	1	4D	5	3B	1	3	1
HAN-5_T05	1	3	8	8	8	———	1	4D	5	———	1	3	1
HAN-5_T06	1	3	8	8	8	1	1	4D	———	3B	1	3	1
HAN-5_T07	1	———	8	8	———	1	1	4D	5	3B	1	———	1

*Nomenclature for the genotype designations (G prefix omitted) follows that described by Suárez et al. (2019b). Results supporting the presence of multiple strains (i.e., detection of >1 genotype) are in bold font. Information on the nucleotide sequences (i.e., signatures) used to identify genotypes are detailed in Table S1, column B, rows 26-134 and 137-140. Underlined values are derived from modified signatures (see Table S1, column B, rows 137-140)*.

### Genomes Assembled

Eight complete and two incomplete HCMV genome sequences were assembled. Complete genome sizes ranged from 235,306 to 235,917 bp. Three genomes presented the complete set of HCMV genes. The remaining seven genomes presented mutations leading to premature termination of at least one gene (most frequently RL5A), a proportion that is in line with previous reports (Sijmons et al., 2015; Suárez et al., 2019b). One genome presented four mutated genes, including UL111A (encoding viral interleukin-10), one genome presented three mutated genes, one genome presented two mutated genes, and the remaining four genomes presented a single mutated gene (Table 3).

**Table 3:** Overview of HCMV genome sequences assembled.

**Patient ID**	**Strain ID**	**Size (bp)**	**Status (no. of gaps)**	Mutations^*^
SYD-1	SYD-SCT1	235,917	Complete	RL5A
SYD-2	SYD-SCT2	235,797	Complete	RL5A
GLA-1	GLA-SOT1	236,114^a^	Incomplete (3)	RL5A, UL1, UL111A, US7
GLA-2	GLA-SOT2	235,742	Complete	None
GLA-3	GLA-SOT3	235,723	Complete	UL1
GLA-4	GLA-SOT4	235,508	Complete	None
HAN-1	HAN-SOT1	235,636	Complete	None
HAN-2	———	———	———	———
HAN-3	HAN-SOT3	234,142^a^	Incomplete (4)	RL6
HAN-4	HAN-SOT4	235,327	Complete	RL6, UL1, UL9
HAN-5	HAN-SOT5	235,306	Complete	RL5A, RL6

* *Mutations leading to premature truncation of the protein*.

a *These assemblies include gaps that might lead to misestimates of the genome size. No genome sequence was assembled from patient HAN-2 due to poor quality of sequencing libraries*.

### Minor Variants and Resistance Mutations

**Figure 3:**
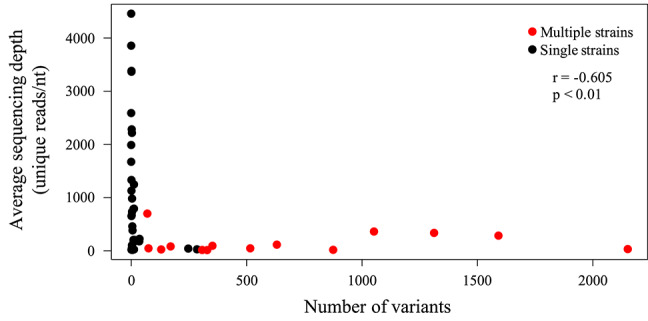
Scatter plot showing the effect of sequencing library diversity and presence of multiple strains on the number of low frequency variants detected. Unique HCMV reads were identified as described in Methods, and the average sequencing depth (reads/nt) of these reads is represented on the Y-axis. The numbers of low frequency (>2%) variants were determined from deduplicated datasets as described in Methods, and are represented on the X-axis. A significant (*p* < 0.01) negative correlation (Spearman) was noted.

**Figure 4:**
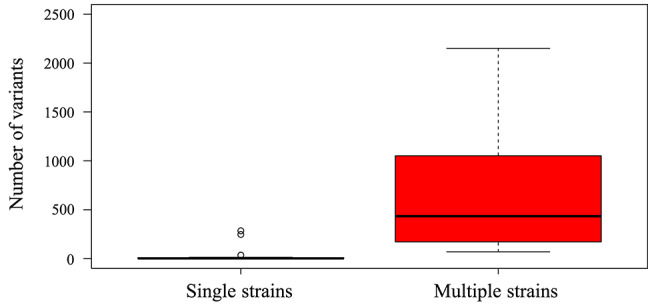
Box-and-whisker plot created using ggplot2 (https://ggplot2.tidyverse.org) showing the number of low frequency (>2%) variants detected by LoFreq in single-strain infections (*n* = 33 datasets) and multiple-strain infections (*n* = 14 datasets). Datasets were classified as single-strain or multiple-strain infections using a genotype-based method described previously (Suárez et al., 2019b). Each box encompasses the first to third quartiles (Q1–Q3) and shows the median as a thick line. For each box, the horizontal line at the end of the upper dashed whisker marks the upper extreme (defined as the smaller of Q3+1.5 [Q3–Q1] and the highest single value), and the horizontal line at the end of the lower dashed whisker marks indicates the lower extreme (the greater of Q1–1.5 [Q3–Q1] and the lowest single value). A significant difference was noted between the number of low frequency variants in libraries with multiple strains and those in libraries with single strains (Mann-Whitney test, *p* < 0.01).

HTS is highly PCR-dependent, which might lead to the incorporation of artifactual variability into sequence datasets. Consequently, only high-quality datasets, that is those with (1) an average sequencing depth of all reads of ≥500 reads/nt, (2) an average sequencing depth of unique reads of ≥10 reads/nt, and (3) ≥95% coverage of the strain Merlin genome, were considered for screening minor variants. In addition, only variants occurring at a frequency of >2% were scored (Xu et al., 2017). Of the 62 sequencing libraries generated, 46 met all quality thresholds. One dataset (SYD-1_T03) met criteria (2) and (3) but exhibited slightly lower average coverage depth (486). With this caveat, this dataset was included in the analysis. The total number of variants across the genome ranged from 0 to 2,151 (Table 4), with low diversity libraries (Figure 3) and multiple-strain infections (Figure 4) showing the highest values.

**Table 4:** Low frequency variants (>2%) in whole genome sequences and in antiviral target genes UL54 and UL97.

**Patient ID**	**Dataset ID^**a**^**	**Variants in whole genome (no.)^**b**^**	**Variants in UL54 (no.)^**b**^**	**Variants in UL97 (no.)^**b**^**	**Strains (no.)^**c**^**
SYD-1	SYD-1_T01	329	8	0	2
	SYD-1_T02	875	20	10	2
	SYD-1_T03	5	0	0	1
	SYD-1_T04	285	4	2	1
	SYD-1_T06	2,151	15	14	2
SYD-2	SYD-2_T01	130	1	0	2
	SYD-2_T02	307	13	3	2
	SYD-2_T03	516	2	1	2
	SYD-2_T04	1,591	5	3	2
	SYD-2_T05	352	2	0	2
	SYD-2_T06	631	8	3	2
	SYD-2_T07	75	2	0	2
	SYD-2_T08	171	3	0	2
	SYD-2_T09	1,312	6	0	2
	SYD-2_T10	1,052	2	1	2
GLA-1	GLA-1_T01	2	0	0	1
	GLA-1_T02	2	0	0	1
	GLA-1_T03	4	0	0	1
	GLA-1_T04	12	0	0	1
	GLA-1_T05	10	0	0	1
GLA-2	GLA-2_T01	1	0	0	1
	GLA-2_T02	1	0	0	1
	GLA-2_T03	1	0	0	1
	GLA-2_T04	12	0	0	1
GLA-3	GLA-3_T01	1	0	0	1
	GLA-3_T02	0	0	0	1
	GLA-3_T03	0	0	0	1
	GLA-3_T04	3	0	0	1
	GLA-3_T05	70	0	0	2
	GLA-3_T06	36	1	0	1
	GLA-3_T07	14	0	0	1
	GLA-3_T08	12	0	0	1
GLA-4	GLA-4_T01	0	0	0	1
	GLA-4_T02	10	0	0	1
	GLA-4_T03	1	0	0	1
	GLA-4_T04	4	0	0	1
	GLA-4_T05	0	0	0	1
	GLA-4_T06	0	0	0	1
	GLA-4_T07	35	2	0	1
	GLA-4_T08	247	8	0	1
HAN-1	HAN-1_T01	10	0	0	1
	HAN-1_T02	3	0	0	1
HAN-4	HAN-4_T05	1	0	0	1
HAN-5	HAN-5_T01	6	0	0	1
	HAN-5_T02	5	0	0	1
	HAN-5_T03	2	0	0	1
	HAN-5_T04	2	0	0	1

a *Results are provided for high-quality datasets (see definition in Results)*.

b *Low frequency variants, including synonymous and non-synonymous mutations, were detected in deduplicated datasets (see Methods)*.

c *Strain enumeration based on the genotyping method described in Suárez et al. (2019b)*.

Mutations in the UL97 gene that are known to confer resistance to ganciclovir and valganciclovir were fixed (i.e., present in 100% of the population) in the assembled genomes and at all time points from two solid organ transplant patients, HAN-4 (A594V) and HAN-5 (L595S) (Table 5). The remaining seven solid organ transplant patients did not present resistance mutations in either the UL97 or the UL54 gene. The assembled genomes from both allogeneic hematopoietic stem cell transplant patients presented mutations at low levels (<25%) in the UL54 gene that are known to confer resistance to foscarnet and ganciclovir (patient SYD-1: A809V; and patient SYD-2: V715M, V781I, A809V, and T838A) (Table 6). However, only three of these mutations were observed in samples collected from at least two different time points from patient SYD-2 (V715M from day 166 to 219; V781I at days 177, 205, and 219; and A809V at days 198 and 205), whereas the rest were observed in samples taken at a single time point (Table 6 and Figure 1).

**Table 5:** High frequency non-synonymous variants in genes UL54 and UL97 identified in assembled genomes.

**Patient ID**	**Strain ID**	**Variants in UL54^**a**^**	**Variants in UL97^**a**^**
SYD-1	SYD-SCT1	G349S, L655S, A688V, L1018F, T1122A, V1164A	T75A, Q126L
SYD-2	SYD-SCT2	G678S, G874R, T1122A, V1164A	T75A, Q126L, T659I
GLA-1	GLA-SOT1	D515G, A972V, N1116H, V1164A	D68N, T75A, Q126L, V244I
GLA-2	GLA-SOT2	Q541R, T1122A, N1147S, V1164A	T75A
GLA-3	GLA-SOT3	L655S, S685N, S897L, D898N, V1164A	T75A, Q126L, H469Y
GLA-4	GLA-SOT4	L655S, S685N, S695T, S897L, T1122A, A1158V, V1164A	T75A
HAN-1	HAN-SOT1	L655S, S685N, S897L, D898N, S1128L, V1164A, P1229Q	T75A
HAN-2	———		
HAN-3	HAN-SOT3	G629S, S1146G, V1164A	T75A, T95S, R112C
HAN-4	HAN-SOT4		T75A, **A594V**
HAN-5	HAN-SOT5	F669L, T1122A, V1164A	T75A, **L595S**

a *Identified using mutation resistance analyser (MRA; Chevillotte et al., 2010). Characterized resistance mutations are depicted in bold font. MRA uses strain TB40/E as reference. No results are provided for patient HAN-2 as low quality libraries precluded a genome assembly*.

**Table 6:** Non-synonymous low frequency variants (>2%) in gene UL54.

**Patient ID**	**Position in UL54^**a**^**	**Mutation**	Days after transplant or after HCMV reactivation^*^									
SYD-1			113	131	155	186	449					
	1045	S349G	———	47%	———	———	16%					
	1940	A647V	———	———	———	18%	———					
	1964	S655L	23%	———	———	———	———					
	2426	**A809V**	———	———	———	21%	———					
	2620	G874R	———	25%	———	———	———					
	2687	G896D	———	21%	———	———	———					
SYD-2			44	51	159	166	173	177	184	198	205	219
	50	A17V	16%	———	———	———	———	———	———	———	———	———
	856	V286M	———	———	———	3%	———	———	———	———	———	———
	1049	C350Y	———	———	———	———	———	———	———	———	2%	———
	1814	T605M	———	———	———	2%	———	———	———	———	———	———
	1901	V634A	———	———	———	———	5%	———	———	———	———	———
	2143	**V715M**	———	———	———	6%	14%	14%	13%	20%	7%	16%
	2156	A719V	———	———	———	3%	———	———	———	———	———	———
	2242	A748T	———	12%	———	———	———	———	———	———	———	———
	2293	R765C	———	———	———	3%	———	———	———	———	———	———
	2341	**V781I**	———	———	———	———	———	5%	———	———	3%	3%
	2374	R792C	———	———	———	———	———	———	———	6%	———	———
	2426	**A809V**	———	———	———	———	———	———	———	7%	4%	———
	2500	A834T^b^	———	10%	———	———	———	———	———	———	———	———
	2512	**T838A**	———	———	———	———	———	———	———	———	2%	———
	2620	G874R	———	22%	———	———	———	———	———	———	———	———
	3251	A1084V	———	———	———	———	———	———	9%	———	———	———
	3457	P1153S	———	12%	———	———	———	———	———	———	———	———
GLA-4			0^*^	8^*^	9^*^	10^*^	13^*^	17^*^	27^*^	30^*^		
	3364	T1122A	———	———	———	———	———	———	2%	———		
	3457	P1153S	———	———	———	———	———	———	2%	———		

* *Days after HCMV reactivation, which occurred 3 years after transplantation. Known resistance mutations are highlighted in red*.

a *Position refers to that in the UL54 gene of strain Merlin*.

b *Although mutation A834T has not been characterized as a resistance mutation, the variant A834P is Scott et al. (2007)*.

## Discussion

The emergence of resistance mutations is one of the major risks of antiviral therapy after transplantation (Emery and Griffiths, 2000). In addition, the presence of multiple-strain infections has been linked to poorer outcomes in transplant recipients (Coaquette et al., 2004; Manuel et al., 2009; Lisboa et al., 2012). Therefore, recognizing the presence of resistance mutations and multiple HCMV strains in patients, even at low levels, would provide potentially important information to clinicians for implementing the most appropriate antiviral therapy. Whole-genome HTS makes it possible to screen for the presence of minor variants (including resistance mutations) and the number of viral strains in a single assay with an unprecedented level of sensitivity. However, a comprehensive assessment of the data is required, as sound interpretation is dependent on understanding and monitoring a range of biological and technological factors (Suárez et al., 2019b).

Here, the dynamics of HCMV genome variation were examined in 11 transplant recipients, including solid organ and allogeneic hematopoietic stem cell transplant recipients. As demonstrated in recent studies (Cudini et al., 2019; Suárez et al., 2019b), the presence of multiple strains in clinical samples (i.e., multiple-strain infections) results in gross overestimation of HCMV genome variability within an individual host, as the variants detected almost entirely reflect genetic differences between the strains present rather than intrahost evolution of any single strain. This conclusion was supported in the present study by the much lower level of genome variability observed in confirmed single-strain infections. In addition, sequencing libraries with low diversity, which were usually derived from samples with low viral loads, also presented a high number of variants, possible due to the prolific incorporation of PCR errors. These findings highlight the importance of ensuring sound interpretation of data, particularly as HTS becomes more widely applied to genotypic resistance testing. It is notable that far more progress in applying HTS in the clinical setting has been achieved in the HIV field, and yet numerous quality control issues remain (Weber et al., 2019). In this study, we opted for a conservative approach in assessing the data, by only analyzing datasets meeting specific quality criteria, and scoring variants at a frequency of >2%. In this regard, further studies focused on evaluating accurate quality thresholds, assessing detection limits, and exploring additional sources of misinterpretation are needed in order to allow HTS technology to be applied confidently to HCMV in the clinical setting.

As observed in this and previous studies (Hage et al., 2017; Cudini et al., 2019; Suárez et al., 2019a,b), the HCMV genome is highly stable in patients over time and during different reactivation episodes (patients HAN-1, HAN-2, and HAN-3), as indicated by the low number of variants detected in longitudinal samples from single-strain infections (Figure 4). This finding is consistent with the expression of a proof-reading DNA polymerase by HCMV (Nishiyama et al., 1983), and contrasts with previous studies characterizing HCMV as a fast-evolving virus that readily generates variants in an individual host at a frequency approaching that of RNA viruses such as HIV (Renzette et al., 2013). These discrepancies, which may have important clinical implications, may be explained by differences in the sequencing technologies used, the analytical approaches taken, and the clinical conditions analyzed.

The occurrence of infections involving multiple HCMV strains may disrupt genome stability by providing opportunities for recombination (Rasmussen et al., 2003; Sijmons et al., 2015; Suárez et al., 2019b). Previous studies have demonstrated that multiple-strain infections are common in allogeneic hematopoietic stem cell and solid organ transplant recipients and are associated with a worse outcome, including a higher prevalence of HCMV disease and a higher rate of graft rejection. Both allogeneic hematopoietic stem cell transplant recipients analyzed in the present study presented multiple-strain (*n* = 2) infections that may have resulted from the positive serological status of both donor and recipient in each case or from the higher level of immunosuppression that such patients typically undergo in comparison to solid organ transplant recipients. However, in order to derive an accurate incidence of multiple-strain infections in transplantation, further studies are warranted that would increase the sample size and represent the various combinations of the serological status of the donor and recipient in both solid organ and allogeneic hematopoietic stem cell transplant cases.

Antiviral drugs may also promote disruption of genome stability by stimulating the selection of variants in genes that render the virus susceptible to those drugs. Approximately 40% of the cases analyzed in the present study developed resistance mutations at various levels in UL54 and UL97. The HCMV genomes sequenced from two solid organ transplant cases (patients HAN-4 and HAN-5) contained fixed mutations in UL97 (A594V and L595S, respectively) that confer resistance to ganciclovir and valganciclovir. This finding is in line with a previous report that the category of solid organ transplants involving a seropositive donor and a seronegative recipient (D+/R–), to which these two cases belonged, account for the highest incidence of resistance (Lurain and Chou, 2010). These mutations were also detected in these patients by Sanger sequencing (Figure 1), but only at specific time points following a recommended procedure (i.e., when the viral load had persisted or increased after 2 weeks of antiviral therapy). However, these mutations stayed fixed in the viral population, even after a change of therapy (patient HAN-5). No resistance mutations were detected in the remaining seven solid organ transplant patients, even though these included three additional D+/R- cases. The incidence of resistance mutations obtained in the present study may not necessarily reflect the general situation, as the number of cases analyzed in this study was limited and included some cases (patients HAN-4 and HAN-5) with previously recognized antiviral resistance mutations detected by Sanger sequencing. In addition, our findings might also have been influenced by the generation of a number of low quality sequencing libraries, such as for patients HAN-2 and HAN-3, or by a shorter time frame of sample collection, such as for the GLA patients (maximum time frame of 31 days between samples). The latter factor is particularly relevant because detection of resistance mutations, except in the immunodeficient pediatric population (Wolf et al., 1998; Houldcroft et al., 2016), is unusual during the first 6 weeks of antiviral therapy (Springer et al., 2005; Lurain and Chou, 2010).

In the present study, both allogeneic hematopoietic stem cell transplant recipients presented resistance mutations (V715M, V781I, A809V, and T838A) at a low level (<25%) in UL54. Mutation V715M, which confers resistance to foscarnet (Baldanti et al., 1996), was detected in patient SYD-2 by Sanger sequencing at day 114 post-transplant after a period of 41 days receiving this antiviral drug. However, this method failed to detect the same mutation 52 days later, as it was present at a low level (6%) (Figure 1 and Table 6). Indeed, this mutation was never cleared despite the change of therapy to ganciclovir or cidofovir. There are several possible explanations for the resilience of this mutation, including the administration of a suboptimal drug dosage, the presence of compensatory mutations in a different region of the HCMV genome, and host factors associated with the level of immunosuppression. Also, in the same patient, mutation V781I, which confers resistance to foscarnet (Cihlar et al., 1998) and ganciclovir (Chou, 2011), was first detected by HTS at 205 days after transplantation at a frequency of <5%, soon after initiation of cidofovir. This mutation was also detected by Sanger sequencing 25 days later (a time point at which no HTS data were available), after completion of cidofovir treatment and initiation of ganciclovir administration. In contrast, mutation A809V, which confers resistance to ganciclovir and foscarnet (Chou et al., 2007), was detected by HTS at 198 days after transplantation and remained for 7 days until being effectively cleared with the initiation of cidofovir. The presence of multiple resistance mutations simultaneously in the same patient, even at low frequency, is of particular interest, as these mutations are unlikely to be detected by traditional non-HTS methods (i.e., Sanger sequencing). Multiple resistance mutations occurring at sub-fixation levels (i.e., present in <100% of the viral population) can lead to a drug resistant phenotype (Chou et al., 2014), a phenomenon that has been observed in fatally immunocompromised pediatric patients (Houldcroft et al., 2016) and solid organ transplant patients (Guermouche et al., 2020). In such circumstances, novel HCMV-specific cell therapies may offer an alternative intervention for patients with clinically resistant disease, especially those found to have clinically resistant HCMV associated with known resistance mutations (Withers et al., 2017). These findings highlight the importance of monitoring resistance mutations with high sensitivity during the course of an antiviral regime, as those present at low level may become prevalent at later stages due to a change to a regime that selects them.

A number of other non-synonymous mutations in UL54 and UL97 were also detected in the cohort. Their capacity to contribute to antiviral resistance is not known and is worthy of further investigation. Moreover, we focused on resistance mutations in UL54 and UL97 as these are the target genes of the antivirals used in the cases analyzed. The application of alternative drugs (Griffiths, 2019), targeting different loci, such as maribavir (UL27) and letermovir (the terminase complex consisting of UL56 and UL89), the potential release of new antiviral drugs acting at other essential HCMV genes, and the possible development of compensatory mutations in other genomic regions, indicates the usefulness of an assay capable of detecting mutations throughout the whole genome, such as the one applied in this study.

## Conclusions

Improved treatment of HCMV-infected transplant patients would benefit from more effective monitoring of host and viral determinants. In this regard, HTS provides an opportunity to carry out comprehensive analyses of the evolution of resistance mutants and other minor variants in the HCMV populations present in transplant recipients. The stability of the viral genome in such patients may be perturbed by the application of antiviral drugs or the presence of multiple strains. Monitoring the development of resistance mutations, even at a low frequency, is potentially of use in assisting clinicians to implement the most appropriate antiviral therapy at a time when it is most likely to be effective. However, it is necessary to have a firm understanding of the data, in terms of its quality and the ways in which it is analyzed, in order to ensure that the conclusions are sound and meaningful.

## Data Availability Statement

The datasets generated for this study were deposited in the European Nucleotide Archive (ENA; project no. PRJEB36759), and the annotated consensus genome sequences were deposited in GenBank (Accession Nos.: MT044476-MT044485).

## Ethics Statement

The studies involving human samples were collected with the approval of: The University of Sydney human research ethics committee (reference 2014/440), National Health Service research Scotland Greater Glasgow and Clyde biorepository (reference 405), and The Institutional review boards of Hannover Medical School (reference 2527-2014). Written informed consent for participation was obtained for samples collected in the University of Sydney. The samples analysed from Glasgow and Hanover were derived from an anonymised archive of samples. Written informed consent for participation was not required for this study in accordance with the national legislation and the institutional requirements.

## Author Contributions

NS and AJD contributed to the conception and design of the study. EB, KL, TG, TS, RG, and DG recruited subjects. NS, EB, KL, TG, SA, BW, SL-H, WG, AD, AH, and BS collected and organized the database. NS wrote the first draft of the manuscript. SC wrote sections of the manuscript. All authors contributed to manuscript revision, read, and approved the submitted version.

## Conflict of Interest

The authors declare that the research was conducted in the absence of any commercial or financial relationships that could be construed as a potential conflict of interest.

## Supplementary Material

Table S1 Information on samples, sequencing libraries, genomes assembled and genotyping results.
